# Single priming and booster dose of ten-valent and 13-valent pneumococcal conjugate vaccines and *Streptococcus pneumoniae* colonisation in children in South Africa: a single-centre, open-label, randomised trial

**DOI:** 10.1016/S2352-4642(23)00025-1

**Published:** 2023-05

**Authors:** Courtney P Olwagen, Alane Izu, Eleonora A M L Mutsaerts, Lisa Jose, Anthonet Koen, Sarah L Downs, Lara Van Der Merwe, Matt Laubscher, Amit J Nana, Andrew Moultrie, Clare L Cutland, Jeffrey R Dorfman, Shabir A Madhi

**Affiliations:** a South Africa Medical Research Council Vaccines and Infectious Diseases Analytics Research Unit, University of the Witwatersrand, Johannesburg, South Africa; b Infectious Diseases and Oncology Research Institute, University of the Witwatersrand, Johannesburg, South Africa; c Faculty of Health Science, and African Leadership in Vaccinology Expertise, University of the Witwatersrand, Johannesburg, South Africa; d Department of Pediatrics, Amsterdam University Medical Center, Amsterdam, Netherlands; e Department of Medical Virology, Department of Pathology, Stellenbosch University, Cape Town, South Africa

## Abstract

**Background:**

Pneumococcal conjugate vaccine (PCV) immunisation has reduced vaccine-serotype colonisation and invasive pneumococcal disease in South Africa, providing the opportunity to consider transitioning from a two-dose (2 + 1) to one-dose (1 + 1) primary series and a booster dose.

**Methods:**

In this single-centre, open-label, randomised trial done in South Africa, infants aged 35–49 days without HIV infection, without childhood immunisations except for BCG and polio, and with gestation age at delivery of at least 37 weeks of age, a birthweight of at least 2500 g, and weight of at least 3500 g at the time of enrolment were randomly assigned (1:1:1:1:1:1), through block randomisation (block size of 30), to receive a single priming dose of ten-valent PCV (PCV10) or 13-valent PCV (PCV13) at either 6 weeks (6-week 1 + 1 group) or 14 weeks (14-week 1 + 1 group), compared with two priming doses at 6 weeks and 14 weeks (2 + 1 group), followed by a booster dose at 9 months of age in all groups. The primary objective of the trial has been published previously. We report the secondary objective of the effect of alternative doses of PCV10 and PCV13 on serotype-specific *Streptococcus pneumoniae* colonisation at 9 months, 15 months, and 18 months of age and a further exploratory analysis in which we assessed non-inferiority of serotype-specific serum IgG geometric mean concentrations 1 month after the booster (10 months of age) and the percentage of participants with serotype-specific IgG titre above the putative thresholds associated with a risk reduction of serotype-specific colonisation between the 1 + 1 and 2 + 1 groups for both vaccines. Non-inferiority was established if the lower limit of the 95% CI for the difference between the proportion of participants (1 + 1 group *vs* 2 + 1 group) above the putative thresholds was greater than or equal to –10%. All analyses were done in the modified intention-to-treat population, which included all participants who received PCV10 or PCV13 according to assigned randomisation group and for whom laboratory results were available. The trial is registered with ClinicalTrials.gov, NCT02943902.

**Findings:**

1564 nasopharyngeal swabs were available for molecular serotyping from 600 infants who were enrolled (100 were randomly assigned to each of the six study groups) between Jan 9 and Sept 20, 2017. There was no significant difference in the prevalence of overall or non-vaccine serotype colonisation between all PCV13 or PCV10 groups. PCV13 serotype colonisation was lower at 15 months of age in the 14-week 1 + 1 group than in the 2 + 1 group (seven [8%] of 85 *vs* 17 [20%] of 87; odds ratio 0·61 [95% CI 0·38–0·97], p=0·037), but no difference was seen at 9 months (nine [11%] of 86 *vs* ten [11%] of 89; 0·92 [0·60–1·55], p=0·87) or 18 months (nine [11%] of 85 *vs* 11 [14%] of 87; 0·78 [0·45–1·22], p=0·61). Compared with the PCV13 2 + 1 group, both PCV13 1 + 1 groups did not meet the non-inferiority criteria for serotype-specific anti-capsular antibody concentrations above the putative thresholds purportedly associated with risk reduction for colonisation; however, the PCV10 14-week 1 + 1 group was non-inferior to the PCV10 2 + 1 group.

**Interpretation:**

The serotype-specific colonisation data reported in this study together with the primary immunogenicity endpoints of the control trial support transitioning to a reduced 1 + 1 schedule in South Africa. Ongoing monitoring of colonisation should, however, be undertaken immediately before and after transitioning to a PCV 1 + 1 schedule to serve as an early indicator of whether PCV 1 + 1 could lead to an increase in vaccine-serotype disease.

**Funding:**

The Bill & Melinda Gates Foundation.

**Research in context**

**Evidence before this study**

Pneumococcal conjugate vaccines (PCVs) remain the most expensive vaccine in routine childhood immunisation programmes. To circumvent costs, considerations are underway to reduce the dosing schedule from a 2 + 1 to a 1 + 1 schedule. The UK became the first country to do so. Careful considerations are needed before transitioning to a reduced dose of PCV in low-income and middle-income countries (LMICs), where residual transmission of vaccine serotypes and the burden of pneumococcal disease remains higher than in high-income countries, such as the UK. To investigate the direct effect of the alternative ten-valent PCV (PCV10) or 13-valent PCV (PCV13) schedules on the serotype-specific pneumococcal carriage, we did a systematic literature review using PubMed to identify clinical studies published in English up to Oct 17, 2022, using the search terms “pneumococcal conjugate vaccines” or “PCV10” or “PCV13”, and “dosing schedule” or “alternate dosing schedule” or “reduced dosing schedule”, and “carriage” or colonization”, and “pneumococcus” or “*Streptococcus pneumoniae*”, “randomized control trial”, “clinical study”. Notably, few data were available, with only a single study comparing 3 + 0 to a 2 + 1 PCV10 dosing schedule in children in Nepal. We found no published studies comparing a 1+1 schedule with a 2 + 1 schedule for either vaccine formulation, although clinical trials are currently underway in The Gambia, India, and Vietnam, and the findings from these studies are expected to be made available in the next few years.

**Added value of this study**

To our knowledge, our study is the first to analyse new acquisition and carriage of vaccine serotypes and non-vaccine serotypes following the booster dose of PCV in children receiving a single priming dose compared with those who received two priming doses of PCV10 or PCV13. The study shows no difference in the acquisition of new vaccine or non-vaccine serotypes between the study groups for both vaccine formulations. We also report lower prevalence of vaccine serotype colonisation post booster in the PCV13 group that received a priming dose at 14 weeks of age and booster at 9 months of age compared with the 2 + 1 group, which received priming doses at 6 weeks and 14 weeks of age and a booster at 9 months of age, particularly for serotype 19F (2·9-fold lower). These results mitigate concerns of a delayed primary series (at 14 weeks only) increasing the incidence of vaccine serotype carriage acquisition and risk of invasive pneumococcal disease in young infants, in whom the first carriage episode can occur within the first few weeks of life in LMICs. Transitioning to a PCV13 1 + 1 dosing schedule, with the priming dose at 14 weeks, in South Africa could translate to better protection against 19F colonisation and the risk of 19F invasive pneumococcal disease in settings such as ours.

**Implications of all the available evidence**

PCV immunisation must remain cost-effective in a mature immunisation programme in places where the risk of pneumococcal disease is already reduced at a population level. The serotype-specific colonisation data reported in this study, together with the primary immunogenicity endpoints of the control trial, support transitioning to a reduced 1 + 1 schedule in South Africa. This will make PCV procurement more affordable, with little risk of increased acquisition of vaccine serotypes, which are usually the most prevalent, invasive, and antibiotic-resistant types in children. Ongoing surveillance of colonisation trends will be necessary as an early indicator of whether transitioning to a 1 + 1 schedule might lead to an increase in the risk of vaccine-serotype disease.

## Introduction

The main pneumococcal conjugate vaccine (PCV) formulations currently in use globally are the ten-valent PCV (PCV10; Synflorix, GlaxoSmithKline, Brentford, UK) and 13-valent (PCV13; Prevenar-13, Pfizer, New York, NY, USA). Both vaccine formulations are efficacious and effective against vaccine-serotype invasive pneumococcal disease and all-cause pneumonia hospitalisation in high-income, middle-income, and low-income settings where widespread immunisation has been implemented.^1^, ^2^, ^3^, ^4^ PCVs were initially licensed as a three-dose primary series with a booster dose in the second year of life (3 + 1 dosing schedule). The high cost of these vaccines, coupled with immunogenicity and epidemiological data, resulted in many countries, including South Africa, adopting a two-dose primary series followed by a booster dose (2 + 1 dosing schedule).^5^ South Africa transitioned from using a seven-valent PCV (PCV7; introduced in April, 2009) to PCV13 in May, 2011, using a schedule of 6 weeks, 14 weeks, and 40 weeks of age (2 + 1 dosing schedule). Despite this reduced dosing schedule, PCV immunisation remains costly, and the sustainability of funding for its inclusion in immunisation programmes of low-income and middle-income countries (LMICs) remains a concern. In South Africa, the cost of procurement of PCV (US$20 per dose) accounts for almost 45% of the total cost of all vaccines purchased for the national Expanded Program on Immunisation (EPI).^6^ Transitioning to a 1 + 1 dosing schedule after vaccine-serotype carriage (>80% reduction) and disease (>90% reduction) have been sufficiently reduced could reduce procurement costs and assist in rationalising the number of injectable vaccines given during early childhood.^7^

In the UK, PCV13 serotype colonisation and disease declined by more than 95% after the introduction of PCV into the public immunisation programme, allowing for the transition from a 2 + 1 to a 1 + 1 dosing schedule in January, 2020.^8^ One of the concerns of transitioning to a reduced 1 + 1 dosing schedule is the potential risk of rebound vaccine-serotype nasopharyngeal colonisation acquisition and consequent increase in pneumococcal disease. This is particularly pertinent in settings where there remains a modest prevalence of residual vaccine-serotype colonisation up to a decade after the introduction of PCVs into routine childhood immunisation programmes.^9^ The prevalence of PCV13 colonisation in children younger than 5 years was 21·9% in 2017, mainly dominated by serotype 19F colonisation (11·6%), in South Africa.^10^ In 2021, the colonisation prevalence of PCV13 in children younger than 5 years was 13·4% (unpublished).

We did an open-label, randomised controlled trial in South Africa, in which children received PCV10 or PCV13 in a 2 + 1 schedule (priming doses at 6 weeks and 14 weeks of age, and a booster at 9 months of age) or a 1 + 1 schedule (with the first dose given either at 6 weeks or 14 weeks of age, with the booster dose given at 9 months of age). The findings showed non-inferiority in immunogenicity, which was the primary objective, of the 1 + 1 compared with 2 + 1 schedules for PCV10 and PCV13.^11^ An analysis of sero-epidemiological data from immunogenicity (PCV10) studies by Voysey and colleagues^12^ used sero-incidence (increases in serotype-specific IgG not attributed to vaccination) as a proxy for serotype-specific colonisation events and derived vaccine serotype-specific thresholds that were associated with lower rates of subsequent homologous serotype colonisation acquisition following the primary series of PCV10. These putative protective correlates against vaccine-serotype colonisation were approximately 2·15 times higher in low-income and lower-middle-income countries compared with those in high-income or upper-middle-income countries, and were also generally higher than the described serotype-specific correlates of protection against invasive pneumococcal disease.^13^

We report on the secondary study objectives of the randomised controlled trial of PCV10 and PCV13 2 + 1 compared with 1 + 1 schedules in South Africa. This included an analysis of colonisation by vaccine serotypes and non-vaccine serotypes following the booster dose of PCV. Furthermore, serotype-specific serum IgG responses were analysed in relation to the putative thresholds proposed by Voysey and colleagues^12^ as being associated with a risk reduction of homologous-serotype colonisation. We hypothesised that there would be no difference in the colonisation prevalence of overall pneumococcus, non-vaccine serotypes, and vaccine serotypes between the study groups.

## Methods

### Study design and participants

Nasopharyngeal flocked swab (FLOQSwabsTM, Copan Diagnostics, Murrieta, CA, USA) samples and serum were collected from children enrolled in a single-centre, open-labelled, randomised trial to evaluate the non-inferiority of a 1 + 1 compared with a 2 + 1 dosing schedule of PCV10 and PCV13 in Soweto, South Africa.^11^ Detailed information on the cohort has been described.^11^ Briefly, healthy infants aged 35–49 days who were born to HIV-uninfected women were enrolled in the study if they did not receive any other childhood immunisations except for BCG and polio at birth, their gestation age at delivery was at least 37 weeks of age, they had a birthweight of at least 2500 g, and they weighed at least 3500 g at the time of enrolment. Exclusion criteria are detailed in the appendix (p 1).

The study was approved by the Human Research Ethics Committee, University of Witwatersrand, Johannesburg, South Africa, and the South African Health Products Regulatory Authority. Written informed consent was obtained from the parents or guardians of all participants at the time of initial enrolment. The protocol is available online.

### Randomisation and masking

Infants were randomly assigned to one of six study groups (1:1:1:1:1:1) through block randomisation (block size of 30) to receive a single dose of PCV10 or PCV13 at 6 weeks of age with a booster at 9 months of age (6-week 1 + 1 group), at 14 weeks of age with a booster at 9 months of age (14-week 1 + 1 group), or two doses at 6 weeks and 14 weeks of age with a booster at 9 months of age (2 + 1 group; days 270 ± 14) in all groups (appendix pp 3–5). This process was implemented by the study statistician using a computer random number generator. In addition, scheduled EPI vaccines were administered to all children enrolled in the study. After consent, study staff assigned each participant a unique study identification number and corresponding randomisation group in sequential order. Parental guardians or clinical staff were not masked to the participant's group assignment; however, all samples were only identified through a unique study identification number and all laboratory personnel were masked to treatment assignment and participant identification throughout the study.

### Procedures

Nasopharyngeal swab samples were collected at 9 months of age (on the same day as, but before, the booster dose was administered), 15 months of age (6 months after the booster), and 18 months of age (9 months after the booster), and were immediately placed in 1 mL skim milk-tryptone-glucose-glycerol (STGG) and transported at 4°C to the Vaccine and Infectious Diseases Analytics Research Unit at the University of the Witwatersrand (Wits VIDA) within 2 h of collection to be stored at −80°C (appendix p 3).

Total nucleic acid was automatically extracted from the transport media of the vortexed nasopharyngeal swab samples using the NucliSens easyMAG extraction system (BioMérieux, Marcy l'Etoile, France) according to the manufacturer's instructions. As an initial step (pre-amplification of DNA) for the Biomark HD system (Standard BioTools [previously known as Fluidigm], San Francisco, CA, USA), specific target amplification (STA) was carried out on extracted DNA, as described elsewhere.^14^, ^15^ Subsequently, quantitative PCR (qPCR) reactions were carried out on the STA products within the 96.96 Dynamic Arrays (Standard BioTools) according to the manufacturer's instructions. Real-time PCR analysis software in the Biomark instrument (Standard BioTools) was used to analyse the qPCR data. Positive samples were defined as those with a cycle of quantification value of at least 36 for each serotype-specific qPCR target and at least three out of the four pneumococcal references genes (*lytA, piaB, ply*, and *xisco*) were detected. The molecular serotyping method (Standard BioTools qPCR) used in this study has been optimised to detect 94 pneumococcal serotypes (59 individual serotypes and 35 serotypes in 16 serogroups), and 15 bacterial pathogens within the nanofluidic real-time qPCR system (appendix p 2).

All reactions included in the Standard BioTools qPCR were effective in amplifying their respective targets with the efficiency of the reactions ranging from 90% to 110%. Within the linear dynamic range, the correlation coefficients of the reactions were higher than 0·98, and all reactions showed high analytical sensitivities (lower limit of detection equivalent <10–100 copies per reaction). Both the inter-assay and intra-assay variations for all respective reactions were less than 0·1 SD, and the accuracy ratio was within 0·1. Standard curves were set up before sample screening and template controls for each reaction were included within each reaction plate. Reaction plates were grouped in batches of ten, and the Cq of controls within each plate was compared with the average cycle of quantification value within the batch and remained within at least 1 quantification cycle variation between standards of each run.

Serotype-specific IgG concentrations were measured for all PCV13 capsular polysaccharides, as detailed previously.^11^ Briefly, serum samples collected 1 month after the booster (10 months of age, 28–35 days after the booster dose of the vaccine was administered) were centrifuged at Wits VIDA laboratories within 4 h of sample collection and then archived at –70°C until assayed (appendix p 2). In-house ELISA was used to measure the serotype-specific IgG concentrations following WHO recommendations.^16^

### Outcomes

The primary study objectives for this study have been published elsewhere.^11^ We report on the secondary study objective of the trial that evaluated the effect of alternative doses of PCV10 and PCV13 on the serotype-specific *Streptococcus pneumoniae* colonisation at 9 months, 15 months, and 18 months of age. We also report on the exploratory study objective of the trial that assessed the non-inferiority of serotype-specific serum IgG geometric mean concentrations and the percentages of children with serotype-specific IgG titres above putative thresholds associated with a risk reduction of serotype-specific colonisation between the 1 + 1 and 2 + 1 groups for both vaccines. The findings from the secondary study objective evaluating the GMCs of serotype-specific antibody concentrations at 18 months of age between the 1 + 1 and 2 + 1 groups for both vaccines will be reported later. Similarly, the exploratory study objective evaluating the vaccine-serotype specific GMC at 18 months of age of differing 1 + 1 dosing schedule compared with the immune response following a 2 + 1 dosing schedule (6 weeks + 14 weeks and booster at 9 months) of the same vaccine formulation (PCV10 or PCV13) will also be reported later.

### Statistical analysis

Participant demographic characteristics were summarised using frequency distributions for categorical variables and means with SDs for continuous variables. The analysis used in this study is part of the pre-planned objectives outlined in the trial protocol and the methods used follow those listed in a prespecified statistical analysis plan. Simple logistic regression analyses were used to compare the nasopharyngeal colonisation prevalence of pneumococcal serotype categories (overall *S pneumoniae*, vaccine serotype, and non-vaccine serotype) between the 1 + 1 and 2 + 1 groups for each vaccine formulation (PCV10 and PCV13) at the time of the booster dose (9 months of age), and at 15 months and 18 months of age. The prevalence of colonisation was calculated as the number of participants with colonisation divided by the total number of participants multiplied by 100. The serotyping method was able to detect concurrent carriage. If a participant had colonisation by multiple serotypes, that participant would contribute to the numerator for each serotype-specific prevalence calculation. For the prevalence of vaccine serotypes, if a participant had colonisation by at least one of the vaccine serotypes, that participant would contribute to the numerator. The calculation was similar for non-vaccine serotypes. As an exploratory analysis, we compared the serotype-specific colonisation prevalence between the 1 + 1 and 2 + 1 groups of each vaccine at each timepoint and the proportion of individuals with antibody concentrations above the putative thresholds of protection from colonisation calculated by Voysey and colleagues.^12^ As the current study focused on addressing both the secondary and exploratory objectives of the study, we did not adjust for multiplicity.

PCV10 vaccine serotypes included serogroups and serotypes 1, 4, 5, 6B, 7A or 7F, 9A or 9V, 14, 18B or 18C, 19F, and 23F, and PCV13 vaccine serotypes included all PCV10 vaccine serotypes and serotypes 3, 6A, and 19A. Serotypes not included in either PCV10 or PCV13 were classified as non-vaccine serotypes for each respective vaccine. New acquisition of a pneumococcal serotype was defined as nasopharyngeal colonisation by a serotype not identified at 9 months of age and identified at 15 months or 18 months of age. The pneumococcal acquisition rates were expressed per 100 children aged 15 months or per 100 children aged 18 months.

The putative protective correlates against PCV10 vaccine-serotype colonisation proposed by Voysey and colleagues^12^ were applied as a proxy for sero-protection in this analysis. Non-inferiority was established if the lower limit of the 95% CI for the difference between the proportion of participants (1 + 1 *vs* 2 + 1) above the putative thresholds was greater than or equal to –10%. All analyses were done in the modified intention-to-treat population, which included all participants who received PCV10 or PCV13 according to assigned randomisation group and for whom laboratory results were available. For the 1 + 1 group to be considered non-inferior to the 2 + 1 group, non-inferiority was required for eight of the ten PCV10 serotypes. Non-inferiority for PCV13 groups was only examined for the PCV10 serotypes because no thresholds were proposed for 3, 6A, and 19A. Two-sided p values of less than 0·05 were considered to be significant. Data were analysed with STATA (version 11.0) and R (version 4.1.1).

The trial was registered with ClinicalTrials.gov, NCT02943902.

### Role of the funding source

The funder of the study had no role in the study design, data collection, data analysis, data interpretation, or writing of the report.

## Results

A total of 1564 nasopharyngeal swabs were available for molecular serotyping from the initial 600 infants enrolled between Jan 9 and Sept 20, 2017 (100 were randomly assigned to each group; appendix pp 4–6). Detailed demographic characteristics of the study cohort have been described previously^11^ (appendix pp 7–8). Briefly, 310 (52%) of 600 children enrolled were male, 290 (48%) were female, and 593 (99%) were Black African. Within each cohort, the distribution of the other demographic characteristics was similar across the study groups.

**Figure 1:**
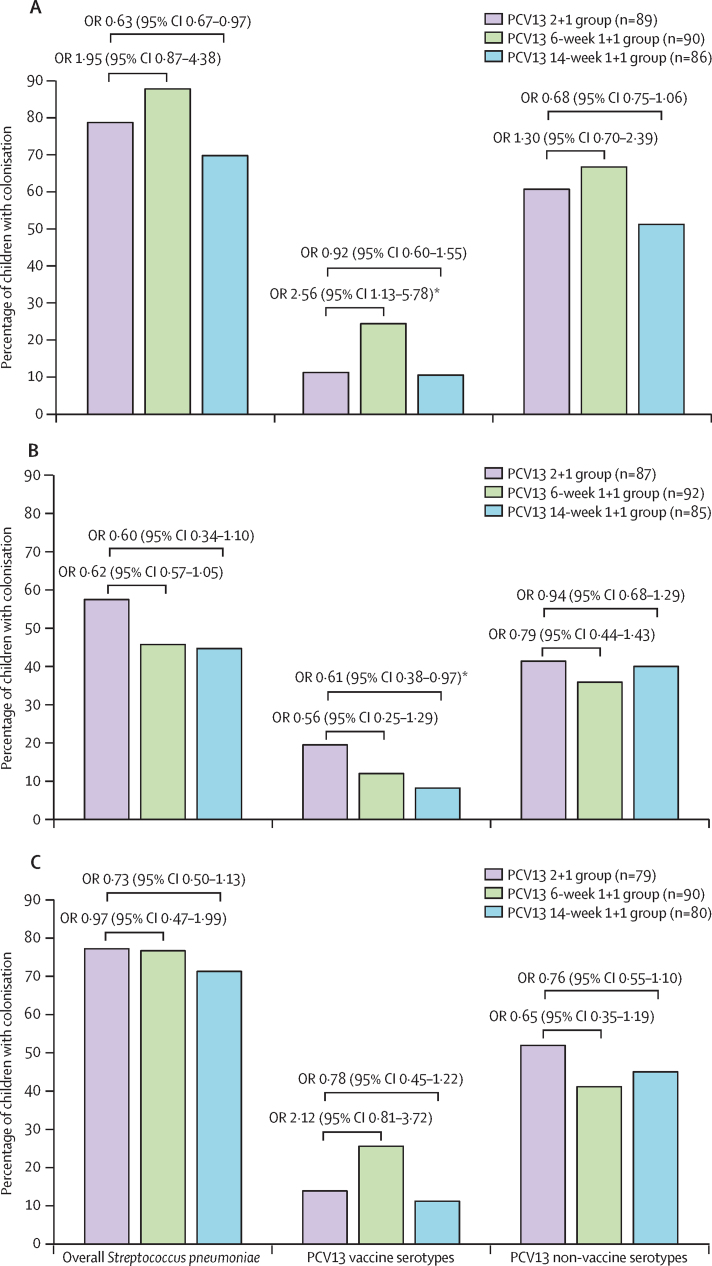
Prevalence of *Streptococcus pneumoniae* colonisation stratified by different PCV13 dosing schedules at 9 months of age (A), 15 months of age (B), and 18 months of age (C) PCV13 vaccine serotypes including serotypes or serogroups 1, 3, 4, 5, 6A, 6B, 7A or 7F, 9A or 9V, 14, 18B or 18C, 19A, 19F, and 23F (differences between serogroups are shown on appendix p 9). Non-vaccine serotypes or serogroups are those that are not included in PCV13 serotypes. ORs and 95% CIs were calculated using logistic regression analyses. OR=odds ratio. PCV13=13-valent pneumococcal conjugate vaccine. *p values <0·05.

Comparing all three PCV13 groups, there was no significant difference at 9 months of age in the prevalence of overall pneumococcal (70–88%) and non-vaccine-serotype (51–67%) colonisation (figure 1A); however, the colonisation prevalence of PCV13 vaccine serotypes was significantly higher in the 6-week 1 + 1 group than in the 2 + 1 group (22 [24%] of 90 *vs* ten [11%] of 89; OR 2·56 [95% CI 1·13–5·78], p=0·024). At 15 months of age (6 months after the booster), the prevalence of PCV13 vaccine-serotype colonisation was lower in the 14-week 1 + 1 group than in the 2+1 group (seven [8%] of 85 *vs* 17 [20%] of 87; OR 0·61 [95% CI 0·38–0·97], p=0·037; figure 1B), particularly for serotype 19F (four [5%] *vs* 12 [14%]; p=0·050; 2·9-fold decrease; appendix p 9). There were no significant differences in grouped PCV13 vaccine serotypes or 19F colonisation between the 6-week 1 + 1 group compared with the 2 + 1 group at 15 months or 18 months of age (figure 1A–C; appendix p 9). Additionally, the prevalence of overall *S pneumoniae* and non-vaccine serotype colonisation was similar between the PCV13 1 + 1 groups and the 2 + 1 group at 15 months or 18 months of age (figure 1A–C).

There was no significant difference in the incidence of new acquisition for PCV13 vaccine serotypes at 15 months or 18 months of age in the 2 + 1 group (13·2 per 100 children at 15 months of age and 11·7 per 100 children at 18 months of age) compared with both the 14-week 1 + 1 group (6·7 per 100 children and 10·6 per 100 children) and the 6-week 1 + 1 group (6·1 per 100 children and 18·0 per 100 children; appendix p 10). Serotype 19F had the highest acquisition rate (per 100 children-years) between 9 months and 18 months of age, but it was not significantly different in the 14-week 1 + 1 group (6·2) than the 2 + 1 group (13·9; p=0·12) or in the 6-week 1 + 1 group (7·5) compared with the 2 + 1 group (13·9; p=0·21; appendix pp 9–10). The acquisition rate of non-vaccines serotypes at 15 months or 18 months of age was similar in all groups and ranged from 20·0 per 100 children to 40·0 per 100 children (appendix p 10).

**Figure 2:**
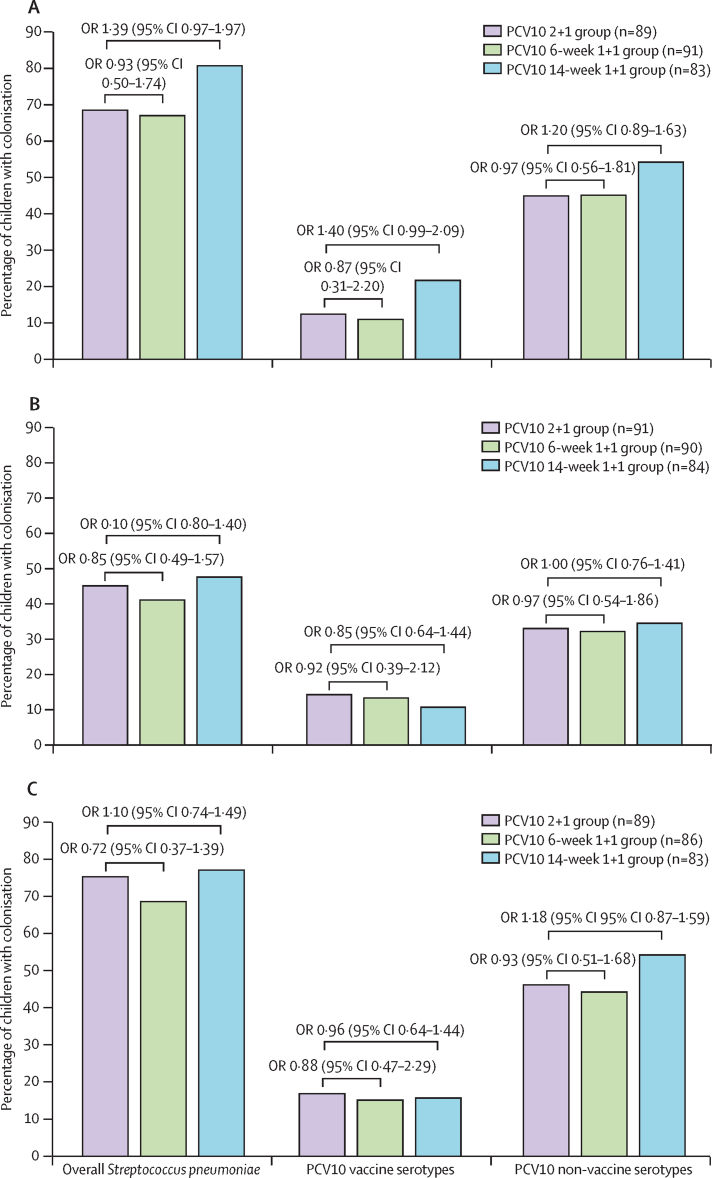
Prevalence of *Streptococcus pneumoniae* colonisation stratified by different PCV10 dosing schedules at 9 months of age (A), 15 months of age (B), and 18 months of age (C) PCV10 vaccine serotypes including serotypes and serogroups 1, 4, 5, 6B, 7A or 7F, 9A or 9V, 14, 18B or 18C, 19F, and 23F (differences between serogroups shown on appendix p 12). PCV10 non-vaccine serotypes or serogroups that are not included in PCV10 serotypes. ORs and 95% CIs were calculated using logistic regression analyses. OR=odds ratio. PCV10=ten-valent pneumococcal conjugate vaccine.

At 15 months of age, the prevalence of overall *S pneumoniae* (41–48%), PCV10 non-vaccine serotype (32–35%), and PCV10 vaccine serotypes (11–14%) were similar across the three PCV10 groups (figure 2A–C). Colonisation prevalence was also similar at 18 months of age across all PCV10 groups for overall *S pneumoniae* (69–77%), PCV10 non-vaccine serotypes (44–54%), and PCV10 vaccine serotypes (15–17%; figure 2A). Serotype 19F was the dominant PCV10 serotype detected in all PCV10 groups at the ages of 9 months (9−16%), 15 months (7–10%), and 18 months (8–12%; appendix p 12).

There was no significant difference in the incidence of new acquisition of PCV10 vaccine and non-vaccine serotypes across the study groups (appendix p 13). New acquisition of PCV10 vaccine serotypes at 15 months or 18 months of age ranged from 6·5 events per 100 children to 14·7 events per 100 children, and serotype 19F was the most frequently acquired serotype in all groups (1·5–10·9 per 100 children; appendix pp 10, 13). The overall acquisition rate of new non-vaccine serotypes ranged from 18·9 per 100 children to 44·8 per 100 children.

**Figure 3:**
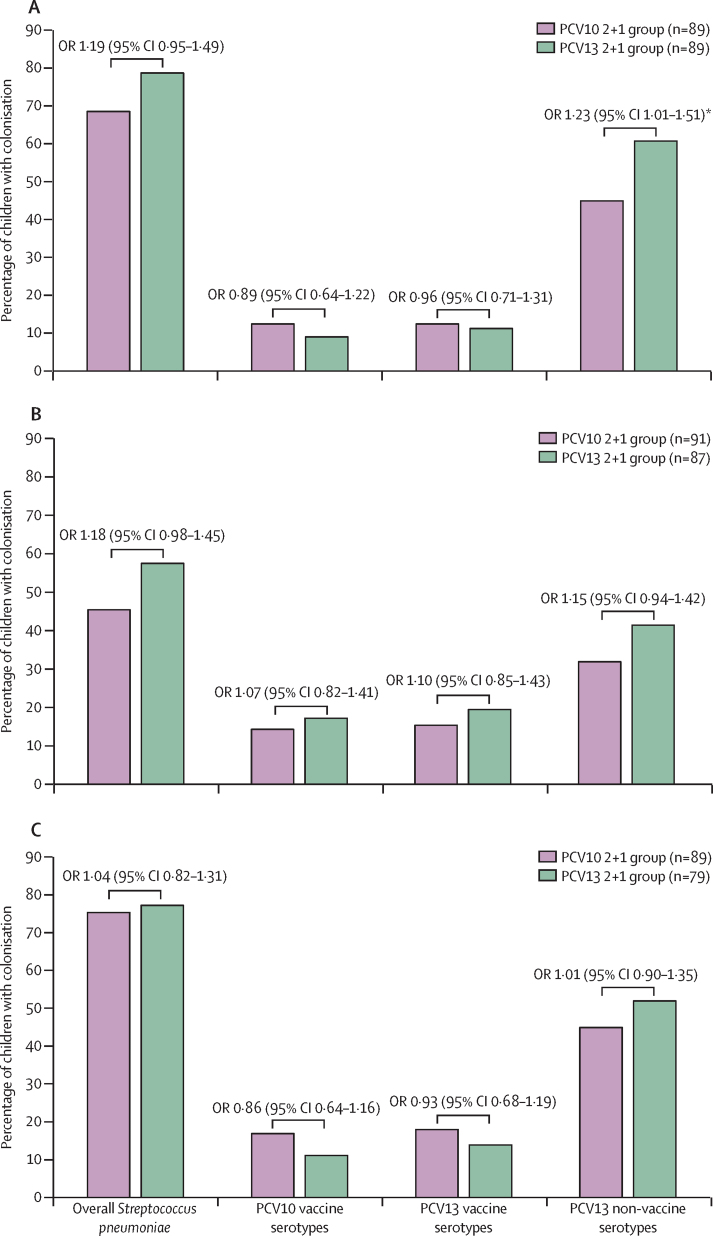
Prevalence of *Streptococcus pneumoniae* colonisation stratified by different PCV formulations at 9 months of age (A), 15 months of age (B), and 18 months of age (C) PCV10 vaccine serotypes including serotypes or serogroups 1, 4, 5, 6B, 7A or 7F, 9A or 9V, 14, 18B or 18C, 19F, and 23F (differences between serogroups are shown on appendix p 14). PCV13 vaccine serotypes including serotypes or serogroups 1, 3, 4, 5, 6A, 6B, 7A or 7F, 9A or 9V, 14, 18B or 18C, 19A, 19F, and 23F. Non-vaccine serotypes or serogroups are those that are not included in PCV13. ORs and 95% CIs were calculated using logistic regression analyses. OR=odds ratio. PCV10=ten-valent pneumococcal conjugate vaccine. PCV13=13-valent pneumococcal conjugate vaccine. *p values <0·05.

We analysed pneumococcal colonisation in children in the 2 + 1 group for either the PCV10 or PCV13 formulation. At 9 months of age (pre-booster dose), the prevalence of overall *S pneumoniae* colonisation (61 [69%] of 89 *vs* 70 [79%] of 89; p=0·13) and PCV13 serotype colonisation (11 [12%] *vs* ten [11%]; p=0·82) was similar between the two groups; however, there was a higher prevalence of PCV13 non-vaccine serotype colonisation in the PCV13 group (54 [61%] *vs* 40 [45%]; OR 1·23 [95% CI 1·01–1·51], p=0·036; figure 3). At 15 months of age, the prevalence of overall *S pneumoniae* colonisation was similar between the PCV13 group (50 [58%] of 87) and PCV10 group (42 [46%] of 91; p=0·10), as was colonisation by PCV13 serotypes (appendix p 14). Serotype 19F was the dominant vaccine serotype detected in PCV10 versus PCV13 groups both before the booster (eight [9%] *vs* seven [8%], p=0·84) and after the booster at 15 months (nine [10%] of 91 *vs* 12 [14%] of 87; p=0·42). The prevalence of serotype 34 at 15 months of age was higher in the PCV13 group (five [6%] of 87]) than in the PCV10 group (0 of 91; p=0·027; appendix p 14).

The percentage of children with serotype-specific capsular IgG geometric mean concentrations above the putative protective correlates against colonisation at 1 month after the booster ranged from 76% to 99% in the 14-week 1 + 1 PCV13 group compared with 76–99% in the 6-week 1 + 1 group and 80–100% in the 2 + 1 PCV13 group (table 1). The 1 + 1 groups did not meet the non-inferiority criteria using the Voysey thresholds.^12^ The proportion above the protective correlates at 1 month after the booster was lower for serotypes 6B, 14, and 23F in the PCV13 6-week 1 + 1 group than in the 2 + 1 group, and lower for serotypes 6B and 18C in the PCV13 14-week 1 + 1 group than in the 2 + 1 group. The percentage of children above the protective correlate associated with a risk reduction in 19F colonisation were 80% (74 of 92) in the 2 + 1 group, 83% (76 of 92) in the 6-week 1 + 1 group, and 89% (79 of 89) in the 14-week 1 + 1 group (table 1).

**Table 1:** Proportion of serum PCV serotype-specific capsular IgG geometric mean concentrations above the putative threshold associated with a risk reduction of serotype-specific colonisation measured 1 month after the booster in children receiving PCV13

	Putative correlate of protection*	**2 + 1 group (n=92)**	**6-week 1 + 1 group (n=92)**	**6-week 1 + 1 *vs* 2 + 1**		**14-week 1 + 1 group (n=89)**	**14-week 1 + 1 *vs* 2 + 1**	
				Difference in proportions of participants (95% CI)	p value†		Difference in proportions of participants (95%CI)	p value‡
1	≥0·81	92 (100%; 96 to 100)	91 (99%; 94 to 100)	−1·09 (−4·81 to 2·69)§	>0·99	88 (99%; 94 to 100)	−1·12 (−4·97 to 2·70)§	0·49
4	≥1·16	88 (96%; 89 to 98)	91 (99%; 94 to 100)	3·26 (−2·46 to 8·84)§	0·37	86 (97%; 91 to 99)	0·98 (−5·56 to 7·41)§	>0·99
5	≥0·73	92 (100%; 96 to 100)	87 (95%; 88 to 98)	−5·43 (−10·93 to 0·30)	0·059	88 (99%; 94 to 100)	−1·12 (−4·97 to 2·70)§	0·49
6B	≥0·5	91 (99%; 94 to 100)	76 (83%; 74 to 89)	−16·30 (−24·66 to −7·25)	0·0001	78 (88%; 79 to 93)	−11·27 (−18·96 to −3·16)	0·0022
7F	≥1·6	90 (98%; 92 to 99)	86 (94%; 87 to 97)	−4·35 (−10·95 to 2·44)	0·27	84 (94%; 88 to 98)	−3·44 (−9·91 to 3·11)§	0·27
9V	≥1·31	88 (96%; 89 to 98)	86 (94%; 87 to 97)	−2·17 (−9·44 to 5·19)§	0·75	83 (93%; 86 to 97)	−2·39 (−9·82 to 5·08)§	0·53
14	≥2·48	84 (91%; 84 to 96)	70 (76%; 66 to 84)	−15·22 (−25·93 to −3·86)	0·0087	78 (88%; 79 to 93)	−3·66 (−13·20 to 5·97)	0·47
18C	≥1·32	82 (89%; 81 to 94)	77 (84%; 75 to 90)	−5·43 (−15·79 to 5·16)	0·39	68 (76%; 67 to 84)	−12·73 (−23·93 to −1·01)	0·029
19F	≥2·54	74 (80%; 71 to 87)	76 (83%; 74 to 89)	2·17 (−9·66 to 13·91)§	0·85	79 (89%; 81 to 94)	8·33 (−2·90 to 19·15)§	0·15
23F	≥0·63	90 (98%; 92 to 99)	82 (89%; 81 to 94)	−8·7 (−16·27 to −0·75)	0·033	83 (93%; 86 to 97)	−4·57 (−11·34 to 2·34)	0·16

Data are n (%; 95% CI) unless otherwise stated. The 6-week 1+1 group received a priming dose at 6 weeks of age and a booster at 9 months of age. The 14-week 1+1 group received a priming dose at 14 weeks of age and a booster at 9 months of age. The 2+1 group received priming doses at 6 weeks and 14 weeks of age and a booster at 9 months of age. PCV=pneumococcal conjugate vaccine. PCV10=ten-valent PCV. PCV13=13-valent PCV.

* Putative correlate of protection against PCV10 serotype-specific colonisation proposed by Voysey and colleagues^12^ was used to define seroprotection in this analysis. No threshold was proposed for serotypes 3, 6A, and 19A, and thus these serotypes are not shown here. Non-inferiority was established if the lower limit of the 95% CI for the difference between the proportion of participants above the putative thresholds was greater than or equal to −10%. For the 1+1 group to be considered non-inferior to the 2+1 group, non-inferiority was required for eight of the ten PCV10 serotypes.

† Comparing proportion of above putative correlate of protection between the 6-week 1+1 group and the 2+1 group.

‡ Comparing proportion of above putative correlate of protection between the 14-week 1+1 group and the 2+1 group.

§ Comparisons that met the non-inferiority criterion.

A similar percentage of children in the PCV10 2 + 1 and both 1 + 1 groups had serotype-specific capsular IgG above the putative correlates proposed by Voysey and colleagues,^12^ albeit lower for serotype 18C in the 6-week 1 + 1 group and higher for serotype 5 in the 14-week 1 + 1 compared with 2 + 1 group (table 2). The 14-week 1 + 1 group was non-inferior to the 2 + 1 group, whereas the 6-week 1 + 1 group did not meet the non-inferiority criteria.

**Table 2:** Proportion of serum PCV serotype-specific capsular IgG geometric mean concentrations above the putative threshold associated with a risk reduction of serotype-specific colonisation measured 1 month after the booster in children receiving PCV10

	Putative correlate of protection*	**2 + 1 group (n=92)**	**6-week 1 + 1 group (n=92)**	**6-week 1 + 1 *vs* 2 + 1**		**14-week 1 + 1 group (n=89)**	**14-week 1 + 1 *vs* 2 + 1**	
				Difference in proportions of participants (95% CI)	p value†		Difference in proportions of participants (95% CI)	p value‡
1	≥0·81	85 (94%; 88 to 98)	87 (94%; 87 to 97)	−0·90 (−8·48 to 6·79)§	>0·99	84 (98%; 92 to 99)	3·23 (−3·50 to 9·73)§	0·44
4	≥1·16	81 (90%; 82 to 95)	90 (97%; 91 to 99)	6·77 (−1·24 to 14·55)	0·078	82 (95%; 89 to 98)	5·35 (−3·18 to 13·56)§	0·25
5	≥0·73	80 (89%; 81 to 94)	83 (89%; 81 to 94)	0·36 (−9·30 to 10·06)§	>0·99	84 (98%; 92 to 99)	8·79 (0·54 to 16·55)§	0·033
6B	≥0·5	88 (98%; 92 to 99)	89 (96%; 90 to 98)	−2·08 (−8·05 to 4·05)§	0·68	83 (97%; 90 to 99)	−1·27 (−7·22 to 4·65)§	0·68
7F	≥1·6	80 (89%; 81 to 94)	80 (86%; 78 to 92)	−2·87 (−12·98 to 7·42)	0·66	79 (92%; 84 to 96)	2·97 (−6·51 to 12·24)§	0·61
9V	≥1·31	82 (91%; 83 to 95)	82 (88%; 80 to 93)	−2·94 (−12·31 to 6·61)	0·63	81 (94%; 87 to 98)	3·07 (−5·46 to 11·39)§	0·57
14	≥2·48	70 (78%; 68 to 85)	69 (74%; 65 to 82)	−3·58 (−16·41 to 9·43)	0·61	76 (88%; 80 to 94)	10·59 (−1·21 to 21·87)§	0·07
18C	≥1·32	82 (91%; 83 to 95)	74 (80%; 70 to 8**7**)	−11·54 (−21·96 to −0·58)	0·036	72 (8%; 75) to 90)	−7·39 (−17·67 to 3·14)	0·17
19F	≥2·54	67 (74%; 64 to 82)	76 (82%; 73 to 88)	7·28 (−5·37 to 19·65)§	0·28	62 (72%; 62 to 81))	−2·35 (−15·96 to 11·31)	0·74
23F	≥0·63	87 (97%; 91 to 99)	83 (89%; 81 to 94)	−7·42 (−15·26 to 0·80)	0·081	83 (97%); 90 to 99)	−0·16 (−6·51 to 6·12)§	>0·99

Data are n (%; 95% CI) unless otherwise stated. The 6-week 1+1 group received a priming dose at 6 weeks of age and a booster at 9 months of age. The 14-week 1+1 group received a priming dose at 14 weeks of age and a booster at 9 months of age. The 2+1 group received priming doses at 6 weeks and 14 weeks of age and a booster at 9 months of age. PCV=pneumococcal conjugate vaccine. PCV10=ten-valent PCV.

* Putative correlate of protection against PCV10 serotype-specific colonisation proposed by Voysey and colleagues^12^ was used to define seroprotection in this analysis. Non-inferiority was established if the lower limit of the 95% CI for the difference between the proportion of participants above the putative thresholds was greater than or equal to −10%. For the 1+1 group to be considered non-inferior to the 2+1 group, non-inferiority was required for eight of the ten PCV10 serotypes.

† Comparing proportion of above putative correlate of protection between the 6-week 1+1 group and the 2+1 group.

‡ Comparing proportion of above putative correlate of protection between the 14-week 1+1 group and the 2+1 group.

§ Comparisons that met the non-inferiority criterion.

## Discussion

Immunisation of children with PCV13 in the 14-week 1 + 1 compared with the 2 + 1 group was associated with a transient reduction in PCV13 colonisation following the booster dose, and particularly a 2·9-fold reduction in 19F colonisation. Serotype 19F is the dominant PCV13 colonising serotype in the era of PCV immunisation in South Africa, as also shown in our study, and elsewhere in Africa;^17^, ^18^, ^19^ and also the major serotype associated with the residual burden of invasive pneumococcal disease from PCV13 serotypes among children receiving routine PCV immunisation.^20^ Furthermore, 19F anti-capsular geometric mean concentrations in the PCV13 14-week 1 + 1 group were higher than in the 2 + 1 group at 1 month after the booster dose,^11^ which could explain the lower prevalence of 19F colonisation after the booster dose in the 14-week 1 + 1 than in the 2 + 1 group. It is plausible that by delaying the primary PCV immunisation dose from 6 weeks to 14 weeks of age, children's immune systems were able to mature, resulting in a higher 19F anti-capsular IgG response and a stronger boost. The decrease in 19F colonisation prevalence was, however, transient, suggesting that 19F anti-capsular IgG antibodies from natural exposure, even in immunised children is short-lived. Nevertheless, transitioning to a 14-week 1 + 1 dosing schedule in our setting could thus translate to better protection against 19F colonisation and consequently could further reduce invasive pneumococcal disease. Furthermore, immunisation of children with PCV13 in the 14-week 1 + 1 compared with the 2 + 1 group was associated with a 2·9-fold reduction in the incidence of new acquisition of 19F from 9 months to 18 months of age. Delayed acquisition or delayed first carriage episode of 19F could also indirectly reduce the risk of disease across other unvaccinated age groups as children are the major transmitters of pneumococcus in communities.^21^, ^22^

The putative correlates of protection, defined by Voysey and colleagues and applied in our study, were determined by a sero-epidemiological analysis of PCV10 immunogenicity studies.^12^ As part of the pre-planned objectives outlined in the trial protocol, we established non-inferiority if the lower limit of the 95% CI for the difference between the proportion of participants (the 1+1 group *vs* the 2+1 group) above the putative thresholds was greater than or equal to –10%.

If we used less stringent criteria to define non-inferiority (ie, by less than 10%), then the PCV13 1 + 1 groups would be non-inferior to the 2 + 1 group. Furthermore, it cannot be ruled out that serum serotype-specific IgG itself might be a proxy for some other mediator of protection against colonisation. Some studies have shown that carriage is not affected by the serotype-specific serum IgG responses,^23^ whereas others have reported that both systemic humoral and cellular immune responses are required to reduce the risk of colonisation.^24^ Nevertheless, the 1 + 1 groups were found to be non-inferior when using the WHO thresholds of 0·35 μg/mL.^11^ Furthermore, our study indicates that there is a low likelihood of an increase in vaccine serotype colonisation in children transitioning to a 1 + 1 dosing schedule, as we reported no increase in the acquisition of vaccine serotypes following a reduced vaccine dose. Although some serotypes for example 18C and 6B did not meet non-inferiority criteria using the Voysey and colleagues' thresholds,^12^ at an individual level, these were associated with a low prevalence of colonisation in our setting. Continued surveillance of changes in *S pneumoniae* colonisation and disease would be warranted when transitioning to a PCV 1 + 1 childhood immunisation schedule in South Africa, where increases in vaccine serotypes such as 18C and 6B could serve as an early indicator for the possible re-emergence and increase in vaccine-serotype pneumococcal disease due to these serotypes.

The study indicates that there might be serotype differences in the effect a 1 + 1 schedule might have on colonisation, as a proxy for disease, and not necessarily that a 1+1 schedule will be inferior to the current 2 + 1 schedule. This is important in the context of higher valency formulations, such as Pfizer's PCV20 (Prevenar-20), which is currently being evaluated in children. If the UK transitions from PCV13 to PCV20, a further consideration arises of whether it is appropriate to remain on the 1 + 1 schedule or return to the 2 + 1 schedule. Serotype-level differences in PCV formulation and schedule demonstrated here indicate that it is not possible to predict the effect of different dosing schedules. In our study, PCV13 administered in a 14-week 1 + 1 schedule seems to be more immunogenic and associated with a lower risk of 19F colonisation compared with the 2 + 1 schedule. Consequently, further head-to-head dosing schedule studies are relevant, including the effect on colonisation by the additional serotypes in PCV20 (8, 10A, 11A, 12F, 15B, 22F, and 33F). In addition to the 2·9-fold reduction in the colonisation prevalence by 19F at 15 months of age, this study showed signals that a reduced PCV13 14-week 1 + 1 dosing schedule might be associated with a reduction in serogroup 11A/D (47% decrease) colonisation and increases in serotype 10A (2-fold increase) and serogroup 15B/C (69% increase) colonisation 6 months after the booster, although changes in these low-prevalence serotypes were not significant.

Our study was limited in that it was not adequately powered to evaluate differences in serotype-specific pneumococcal colonisation between the study groups. For this reason, we did not adjust for multiplicity as the findings address a secondary objective of the study and were hypothesis generating. Larger studies that are adequately powered to assess serotype-level differences are still needed to corroborate our findings. Another limitation of the study was that colonisation was assessed at three timepoints (9 months, 15 months, and 18 months of age), only allowing for analysis of serotype acquisition at 15 months and 18 months of age, and it is plausible that acquisition events outside the sampling period were missed. Last, the Standard BioTools method is unable to differentiate between vaccine serotypes 7F, 9V, and 18C from non-vaccine serotypes 7A, 9A, and 18B, respectively, and colonisation prevalence for these vaccine serotypes might have been overestimated. Also, the method does not directly detect non-typeable pneumococcus, and it is thus possible that a small proportion of pneumococcal positive samples were either non-typeable pneumococcus or a non-detected pneumococcal serotype. Nevertheless, the serotypes not covered by the assay (ie, 7D) are uncommon in carriage.

The findings from this study, together with the experience in the UK, which transitioned to a 1 + 1 schedule, is being considered by the National Advisory Group on Immunization in South Africa to determine whether there should be policy changes to a 1 + 1 schedule in South Africa. Ongoing monitoring of colonisation will, however, be important as a possible early indicator of whether transitioning to a 1 + 1 schedule might inadvertently lead to an increase in vaccine-serotype disease.

## Data sharing

Data used to generate the results reported in this study will be made available following publication to researchers who provide a methodologically sound proposal. Data will only be made available if approval is granted from the Human Research Ethics Committee, University of Witwatersrand, Johannesburg, South Africa. Furthermore, all requesters will need to sign a data transfer agreement. Requests should be directed to the corresponding author.

## Declaration of interests

GlaxoSmithKline (GSK) awarded grant funding to Wits VIDA for research related to PCVs. Furthermore, GSK and Pfizer have funded Wits VIDA to undertake non-pneumococcal research. However, neither Pfizer nor GSK contributed to the funding of this study. JRD has received grants from the Poliomyelitis Research Foundation, consultation fees from the international AIDS Vaccine Initiative, and payment from Sanofi-Aventis South Africa and Sci Mentum for work not related to this manuscript. CPO has received grants from Pfizer and the Bill & Melinda Gates Foundation, payment from Sanofi-Aventis South Africa, and support from the Gates Foundation to attend a meeting unrelated to this work. SAM has received grants from Pfizer, Minervax, GSK, the Gates Foundation, and the South African Medical Research Council, and he has received honoraria and support to attend a meeting from GSK and MSD unrelated to this work. CLC has received grants from Sanofi and Duetsche Gesellschaft fur Internationale Zusammenarbeit. All other authors declare no competing interests.
