# Supplementary material for: Single priming and booster dose of ten-valent and 13-valent pneumococcal conjugate vaccines and Streptococcus pneumoniae colonisation in children in South Africa: a single-centre, open-label, randomised trial
Source: Lancet Child Adolesc Health. 2023 May;7(5):326–35. doi: 10.1016/S2352-4642(23)00025-1 (PMC10127219; doi:10.1016/S2352-4642(23)00025-1)
Supplement: Supplementary appendix [file mmc1.pdf]

THE LANCET
Child & Adolescent Health

Supplementary appendix

This appendix formed part of the original submission and has been peer reviewed. We post it as supplied by the authors.

Supplement to: Olwagen CP, Izu A, Mutsaerts EAML, et al. Single priming and booster dose of ten-valent and 13-valent pneumococcal conjugate vaccines and *Streptococcus pneumoniae* colonisation in children in South Africa: a single-centre, open-label, randomised trial. *Lancet Child Adolesc Health* 2023; published online March 16. https://doi.org/10.1016/S2352-4642(23)00025-1.

Supplementary document: Single priming and booster dose of 10-valent and 13-valent pneumococcal conjugate vaccine (PCV) and *Streptococcus pneumoniae* colonization in South African children: a single-centre, open-labeled, randomized trial

Supplementary text:

1.1. Study population and study exclusion criteria

Infants born at the Chris Hani Baragwanath Academic Hospital (CHBAH) were identified as potential study participants after delivery or when visiting the clinic at 6 weeks of age to receive their routine immunizations. The CHBAH is a public hospital which provides secondary-tertiary level health care situated in Soweto, South Africa. Notably, the study was conducted at the Wits VIDA Research Unit, which is based at the hospital, but enrolments was from the general population of Soweto. Infants were excluded for the study if they had received any investigational vaccine/drug or PCV were not enrolled in the study as well as infants who had been hospitalized previously for any respiratory illness or had a confirmed history of IPD. Other exclusion criteria included infants who had any significant congenital abnormalities, had received a blood transfusion (or other blood products) since birth, had a known vaccine allergy or febrile illness at the time of enrolment, or if any immunodeficiency conditions were suspected. Lastly, if the family planned to relocate to an area outside of the catchment zone within the next 2 years, then the infant was also not enrolled.

1.2. Interventions

During the vaccination visits, infants received either two or three intramuscular injections containing 0.5mL of either PCV10 (Synoflorix ®, GlaxoSmithKline, Rixensart, Belgium) or PCV13 (Prevnar-13®, Pfizer, New York City, United States) in the anterolateral thigh. In addition, scheduled EPI vaccines were administered to all children enrolled in the study. These included combined diphtheria, tetanus, pertussis, hepatitis B, poliomyelitis, and *Haemophilus influenzae* type b conjugate vaccine (Hexaxim®, Sanofi Pasteur, Paris, France) administered at 6, 10, 14 weeks, and 15 months of age, oral rotavirus vaccine (Merieux®, Institut Merieux, Lyon, France) given at 6 and 14 weeks of age, and measles vaccine administered at 6 and 12 months of age (Measbio®, The Biovac Institute, Cape Town, South Africa). After each vaccine was administered, a study doctor or nurse observed the infant for immediate reactions.

Supplementary Table 1: Pneumococcal serotypes and other pathogens detected by the nanofluidic qPCR assay

**Individual pneumococcal serotypes**		**Pneumococcal serotypes within serogroups**	**Bacterial targets**	***Hinfluenzae subtyping***
Serotype 1	Serotype 22A	Serogroup 7A/F	*Streptococcus pneumoniae*	Haemophilus influenzae type B
Serotype 2	Serotype 22F	Serogroup 9A/V	*Haemophilus influenzae*	
Serotype 3	Serotype 23A	Serogroup 9L/N	*Moraxella catarrhalis*	
Serotype 4	Serotype 23B	Serogroup 10 C/F	*Neisseria lactamica*	
Serotype 5	Serotype 23F	Serogroup 11A/D	*Neisseria meningitidis*	
Serotype 6A	Serotype 24A	Serogroup 11B/C	*Staphylococcus aureus*	
Serotype 6B	Serotype 27	Serogroup 12AF/44	*Streptococcus pyogenes*	
Serotype 6C	Serotype 29	Serogroup 15A/F	*Bordetella pertussis*	
Serotype 6D	Serotype 31	Serogroup 15B/C	*Bordetella holmesii*	
Serotype 8	Serotype 33B	Serogroup 18B/C	*Bordetella bronchiseptica*	
Serotype 10A	Serotype 33C	Serogroup 28A/F	*Bordetella parapertussis*	
Serotype 10B	Serotype 33D	Serogroup 24B/F	*Klebsiella pneumoniae*	
Serotype 11E	Serotype 34	Serogroup 25A/F	*Group B streptococcus*	
Serotype 11F	Serotype 35B	Serogroup 32A/F	*Acinetobacter baumanii*	
Serotype 12B	Serotype 35F	serogroup 33A/F	*Escherichia coli*	
Serotype 13	Serotype 36	Serogroup 35AC/42		
Serotype 14	Serotype 37	Serogroup 7BC/40		
Serotype 16A	Serotype 38			
Serotype 16F	Serotype 39			
Serotype 17A	Serotype 41A			
Serotype 18A	Serotype 41F			
Serotype 18F	Serotype 43			
Serotype 19A	Serotype 45			
Serotype 19B	Serotype 46			
Serotype 19F	Serotype 47A			
Serotype 20	Serotype 47F			
Serotype 21	Serotype 48			

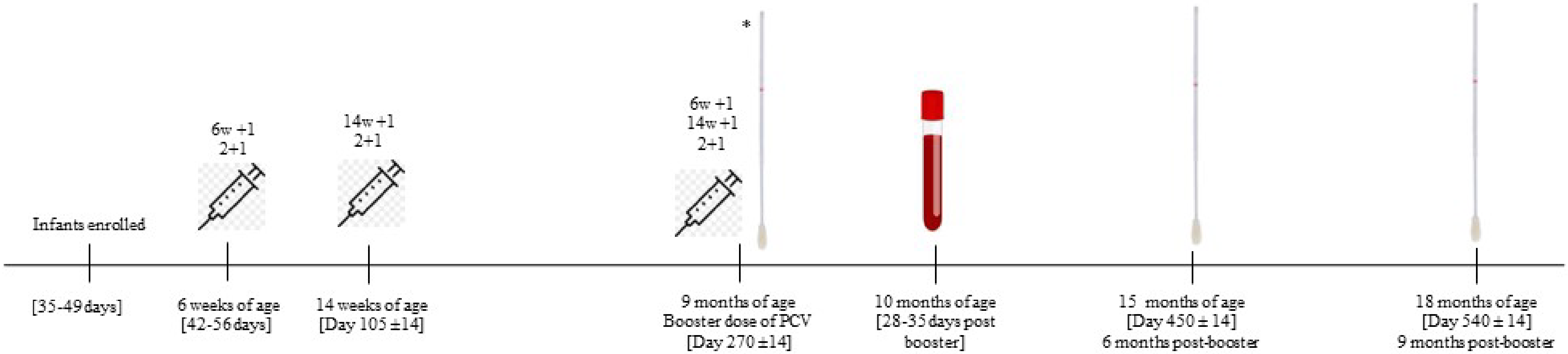

Supplementary Figure 1: Timeline of PCV vaccinations and sample collection

* NP swab sample was taken from all participants on the same day as booster-dose prior to vaccine administration

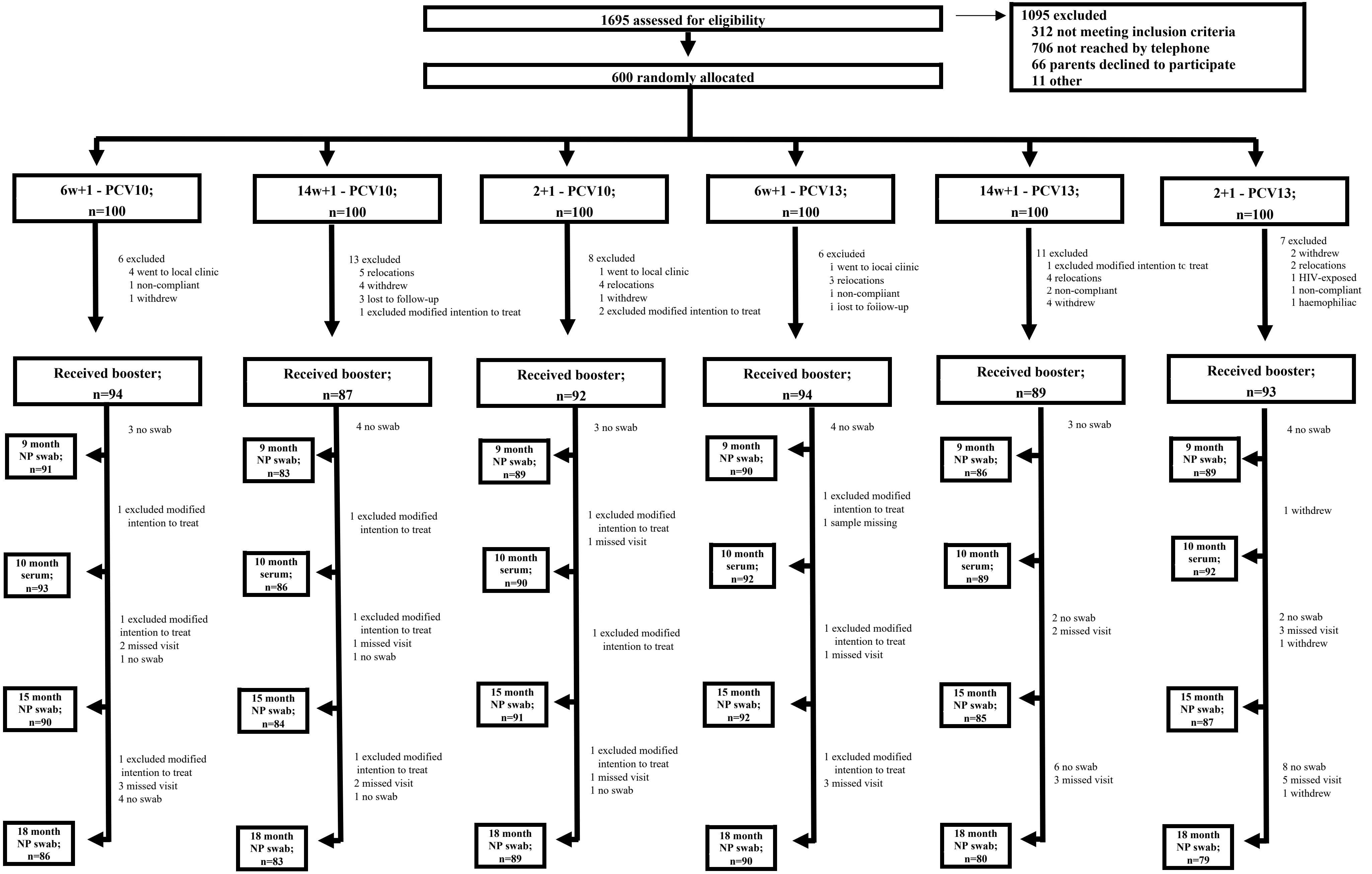
1695 assessed for eligibility
1095 excluded
312 not meeting inclusion criteria
706 not reached by telephone
66 parents declined to participate
11 other
600 randomly allocated
6w+1 - PCV10; n=100
14w+1 - PCV10; n=100
2+1 - PCV10; n=100
6w+1 - PCV13; n=100
14w+1 - PCV13; n=100
2+1 - PCV13; n=100
6 excluded
4 went to local clinic
1 non-compliant
1 withdrew
13 excluded
5 relocations
4 withdrew
3 lost to follow-up
1 excluded modified intention to treat
8 excluded
1 went to local clinic
4 relocations
1 withdrew
2 excluded modified intention to treat
6 excluded
1 went to local clinic
3 relocations
1 non-compliant
1 lost to follow-up
11 excluded
1 excluded modified intention to treat
4 relocations
2 non-compliant
4 withdrew
7 excluded
2 withdrew
2 relocations
1 HIV-exposed
1 non-compliant
1 haemophiliac
Received booster; n=94
Received booster; n=87
Received booster; n=92
Received booster; n=94
Received booster; n=89
Received booster; n=93
3 no swab
4 no swab
3 no swab
4 no swab
3 no swab
4 no swab
9 month NP swab; n=91
9 month NP swab; n=83
9 month NP swab; n=89
9 month NP swab; n=90
9 month NP swab; n=86
9 month NP swab; n=89
1 excluded modified intention to treat
1 excluded modified intention to treat
1 excluded modified intention to treat
1 missed visit
1 excluded modified intention to treat
1 sample missing
1 withdrew
10 month serum; n=93
10 month serum; n=86
10 month serum; n=90
10 month serum; n=92
10 month serum; n=89
10 month serum; n=92
1 excluded modified intention to treat
2 missed visit
1 no swab
1 excluded modified intention to treat
1 missed visit
1 no swab
1 excluded modified intention to treat
1 excluded modified intention to treat
1 missed visit
2 no swab
2 missed visit
2 no swab
3 missed visit
1 withdrew
15 month NP swab; n=90
15 month NP swab; n=84
15 month NP swab; n=91
15 month NP swab; n=92
15 month NP swab; n=85
15 month NP swab; n=87
1 excluded modified intention to treat
3 missed visit
4 no swab
1 excluded modified intention to treat
2 missed visit
1 no swab
1 excluded modified intention to treat
1 missed visit
1 no swab
1 excluded modified intention to treat
3 missed visit
6 no swab
3 missed visit
8 no swab
5 missed visit
1 withdrew
18 month NP swab; n=86
18 month NP swab; n=83
18 month NP swab; n=89
18 month NP swab; n=90
18 month NP swab; n=80
18 month NP swab; n=79

Supplementary Figure 2: Trial profile

Participants were randomly allocated to receive either a single dose of PCV10 or PCV13 at 6 (6w +1) or 14 (14w +1) weeks of age or two doses at 6 and 14 weeks (2+1) of age, with a booster at 9 months of age in all groups. PCV10 = 10-valent pneumococcal conjugate vaccine. PCV13 = 13-valent pneumococcal conjugate vaccine

Supplementary Table 2: Number of available nasopharyngeal swab samples for molecular serotyping stratified by study groups

	PCV10 (14w +1)	PCV10 (6w +1)	PCV10 2+1	PCV13 (14w +1)	PCV13 (6w +1)	PCV13 2+1	Total
**Number of infants enrolled**	100	100	100	100	100	100	600
**Total number of swabs tested**	250	267	269	251	272	255	1564
Visit 1 (9 months of age)	83	91	89	86	90	89	528
Visit 2 (15 months of age)	84	90	91	85	92	87	529
Visit 3 (18 months of age)	83	86	89	80	90	79	507
**Total number of Serum samples tested (10 months of age)**	86	93	90	89	92	92	542

Supplementary Table 3: Demographic characteristics of study participants

	14w+1-PCV10	6w+1-PCV10	2+1-PCV10	14w+1-PCV13	6w+1-PCV13	2+1-PCV13
**9 months of age**						
Number of swabs tested	83	91	89	86	90	89
Mode of delivery: normal vaginal birth (%)	38 (45.8)	47 (51.6)	47 (52.8)	41 (47.7)	45 (50)	40 (44.9)
: Forceps/vacuum delivery (%)	0	1 (1.1)	0	1 (1.2)	0	1 (1.1)
: Emergency caesarean section (%)	38 (45.8)	40 (44)	36 (40.4)	37 (43)	36 (40)	37 (41.6)
: Elective caesarean section (%)	7 (8.4)	3 (3.3)	6 (6.7)	7 (8.1)	9 (10)	11 (12.4)
Gestational age: Term (>=37wks; %)	83 (100)	91 (100)	89 (100)	86 (100)	90 (100)	89 (100)
Sex: Male (%)	37 (44.6)	50 (54.9)	51 (57.3)	39 (45.3)	44 (48.9)	50 (56.2)
: Female (%)	46 (55.4)	41 (45.1)	38 (42.7)	47 (54.7)	46 (51.1)	39 (43.8)
Race: Black (%)	83 (100)	91 (100)	87 (97.8)	85 (98.8)	88 (97.8)	89 (100)
: Coloured (%)	0	0	2 (2.2)	1 (1.2)	2 (2.2)	0
Mean birth weight (SD) in grams	3223 (385)	3283 (429)	3310 (480)	3212 (435)	3226 (484)	3160 (385)
Mean head circumference (SD) in cm at birth	35 (2)	35 (2)	35 (2)	35 (2)	34 (2)	35 (2)
Mean birth length (SD) in cm at birth	50 (2)	51 (3)	51 (3)	50 (3)	50 (4)	50 (3)
Outcome of delivery: Discharged home with mother (%)	82 (98.8)	87 (95.6)	89 (100)	85 (98.8)	87 (96.7)	86 (96.6)
: Discharged home with a family member (mother admitted/died) (%)	0	1 (1.1)	0	0	0	1 (1.1)
: Admitted to neonatal ward (%)	1 (1.2)	3 (3.3)	0	1 (1.2)	3 (3.3)	2 (2.2)
Medical history	2 (2.4)	8 (8.8)	5 (5.6)	8 (9.3)	3 (3.3)	7 (7.9)
Mean age (SD) at the study visit	9 (0.4)	9 (0.1)	9 (0.4)	9 (0.1)	9 (0.1)	9.1 (0.5)
Antibiotic used at study visit (SD)	1 (1.2)	2 (2.2)	0	2 (2.3)	1 (1.1)	4 (4.5)
Mean weight (SD) in KG at the study visit	8.6 (1.2)	8.9 (1.4)	8.8 (1.5)	8.5 (1.3)	8.9 (1.3)	8.6 (1.3)
Mean length (SD) in cm at the study visit	70.9 (3)	71.7 (3.5)	71.8 (3.3)	70.8 (3.1)	71.3 (3.1)	70.8 (2.9)
Respiratory rate (breaths/min) at study visit	36.3 (4.7)	36.7 (5.4)	36.2 (4.7)	35.7 (5.1)	37.4 (5.2)	36.4 (7.1)
Heart rate (beats/min) at study visit	131.7 (8.3)	133.3 (6.5)	130.3 (9.3)	131.1 (8)	132.6 (7.2)	132.7 (7.4)
**15 months of age**						
Number of swabs tested	84	90	91	85	92	87
Mode of delivery: normal vaginal birth (%)	41 (48.8)	48 (53.3)	48 (52.7)	40 (47.1)	46 (50)	40 (46)
: Forceps/vacuum delivery (%)	0	1 (1.1)	0	1 (1.2)	0	1 (1.1)
: Emergency caesarean section (%)	37 (44)	39 (43.3)	36 (39.6)	37 (43.5)	36 (39.1)	36 (41.4)
: Elective caesarean section (%)	6 (7.1)	2 (2.2)	7 (7.7)	7 (8.2)	10 (10.9)	10 (11.5)
Gestational age: Term (>=37wks; %)	84 (100)	90 (100)	91 (100)	85 (100)	92 (100)	87 (100)
Sex: Male (%)	39 (46.4)	51 (56.7)	52 (57.1)	39 (45.9)	46 (50)	51 (58.6)
: Female (%)	45 (53.6)	39 (43.3)	39 (42.9)	46 (54.1)	46 (50)	36 (41.4)
Race: Black (%)	84 (100)	90 (100)	89 (97.8)	84 (98.8)	90 (97.8)	86 (98.9)
: Coloured (%)	0	0	2 (2.2)	1 (1.2)	2 (2.2)	1 (1.1)
Mean birth weight (SD) in grams	3210 (377)	3285 (429)	3313 (475)	3195 (427)	3241 (482)	3161 (385)
Mean head circumference (SD) in cm at birth	35 (2)	35 (2)	35 (2)	35 (2)	35 (2)	35 (2)
Mean birth length (SD) in cm at birth	50 (3)	51 (3)	51 (3)	50 (3)	50 (4)	50 (3)
Outcome of delivery: Discharged home with mother (%)	83 (98.8)	86 (95.6)	91 (100)	84 (98.8)	89 (96.7)	85 (97.7)
: Discharged home with a family member (mother admitted/died) (%)	0	1 (1.1)	0	0	0	1 (1.1)
: Admitted to neonatal ward (%)	1 (1.2)	3 (3.3)	0	1 (1.2)	3 (3.3)	1 (1.1)
Medical history	2 (2.4)	8 (8.9)	5 (5.5)	8 (9.4)	3 (3.3)	7 (8)
Mean age (SD) at the study visit	15.1 (0.3)	15.1 (0.4)	15.1 (0.2)	15.1 (0.1)	15.1 (0.3)	15.1 (0.3)
Antibiotic used at study visit (SD)	1 (1.2)	3 (3.3)	1 (1.1)	2 (2.4)	2 (2.2)	0
Mean weight (SD) in KG at the study visit	10.1 (1.5)	10.5 (1.6)	10.6 (1.7)	10 (1.4)	10.7 (1.7)	10.5 (1.8)
Mean length (SD) in cm at the study visit	77.8 (3.2)	78.2 (3)	77.9 (6.4)	77.8 (3.1)	78.6 (3.2)	78 (3.5)
Respiratory rate (breaths/min) at study visit	33.8 (4.5)	32.8 (4.2)	32.6 (4.4)	33.8 (4.9)	32.8 (4.6)	32.6 (4.4)
Heart rate (beats/min) at study visit	128.1 (8.8)	126.2 (8.6)	127.1 (8.8)	126.9 (9.2)	127 (8.8)	126.8 (8.7)
**18 months of age**						
Number of swabs tested	83	86	89	80	90	79
Mode of delivery: normal vaginal birth (%)	39 (47)	45 (52.3)	47 (52.8)	37 (46.2)	44 (48.9)	36 (45.6)
: Forceps/vacuum delivery (%)	0	1 (1.2)	0	0	0	1 (1.3)
: Emergency caesarean section (%)	37 (44.6)	38 (44.2)	35 (39.3)	36 (45)	36 (40)	32 (40.5)
: Elective caesarean section (%)	7 (8.4)	2 (2.3)	7 (7.9)	7 (8.8)	10 (11.1)	10 (12.7)
Gestational age: Term (>=37wks; %)	83 (100)	86 (100)	89 (100)	80 (100)	90 (100)	79 (100)
Sex: Male (%)	39 (47)	48 (55.8)	52 (58.4)	36 (45)	45 (50)	47 (59.5)
: Female (%)	44 (53)	38 (44.2)	37 (41.6)	44 (55)	45 (50)	32 (40.5)
Race: Black (%)	83 (100)	86 (100)	87 (97.8)	79 (98.8)	88 (97.8)	78 (98.7)
: Coloured (%)	0	0	2 (2.2)	1 (1.2)	2 (2.2)	1 (1.3)
Mean birth weight (SD) in grams	3217 (376)	3276 (433)	3323 (476)	3198 (437)	3230 (475)	3161 (376)

Mean head circumference (SD) in cm at birth	35 (1)	35 (2)	35 (2)	35 (2)	35 (2)	35 (2)
Mean birth length (SD) in cm at birth	50 (3)	50 (3)	51 (3)	50 (3)	50 (4)	50 (3)
Outcome of delivery: Discharged home with mother (%)	82 (98.8)	82 (95.3)	89 (100)	79 (98.8)	87 (96.7)	78 (98.7)
: Discharged home with a family member (mother admitted/died) (%)	0	1 (1.2)	0	0	0	1 (1.3)
: Admitted to neonatal ward (%)	1 (1.2)	3 (3.5)	0	1 (1.2)	3 (3.3)	0
Medical history	2 (2.4)	8 (9.3)	5 (5.6)	8 (10)	3 (3.3)	5 (6.3)
Mean age (SD) at the study visit	18.1 (0.2)	18.5 (3.7)	18.1 (0.3)	18.1 (0.1)	18.1 (0.2)	18.1 (0.2)
Antibiotic used at study visit (SD)	1 (1.2)	0	0	2 (2.5)	1 (1.1)	0
Mean weight (SD) in KG at the study visit	10.7 (1.5)	11.1 (1.6)	11.1 (1.8)	10.7 (1.9)	11.9 (6.8)	11.1 (1.9)
Mean length (SD) in cm at the study visit	80.9 (3.6)	81.3 (3.3)	81.6 (3.5)	80.8 (3.1)	81.7 (3.2)	81 (3.2)
Respiratory rate (breaths/min) at study visit	32.3 (7.4)	32.1 (4.8)	31.4 (4.1)	32.1 (7.4)	31.9 (4)	31.9 (3.9)
Heart rate (beats/min) at study visit	124 (8)	125.6 (8.9)	124.7 (7.8)	126.2 (8.5)	124.9 (8.5)	125.1 (8.6)

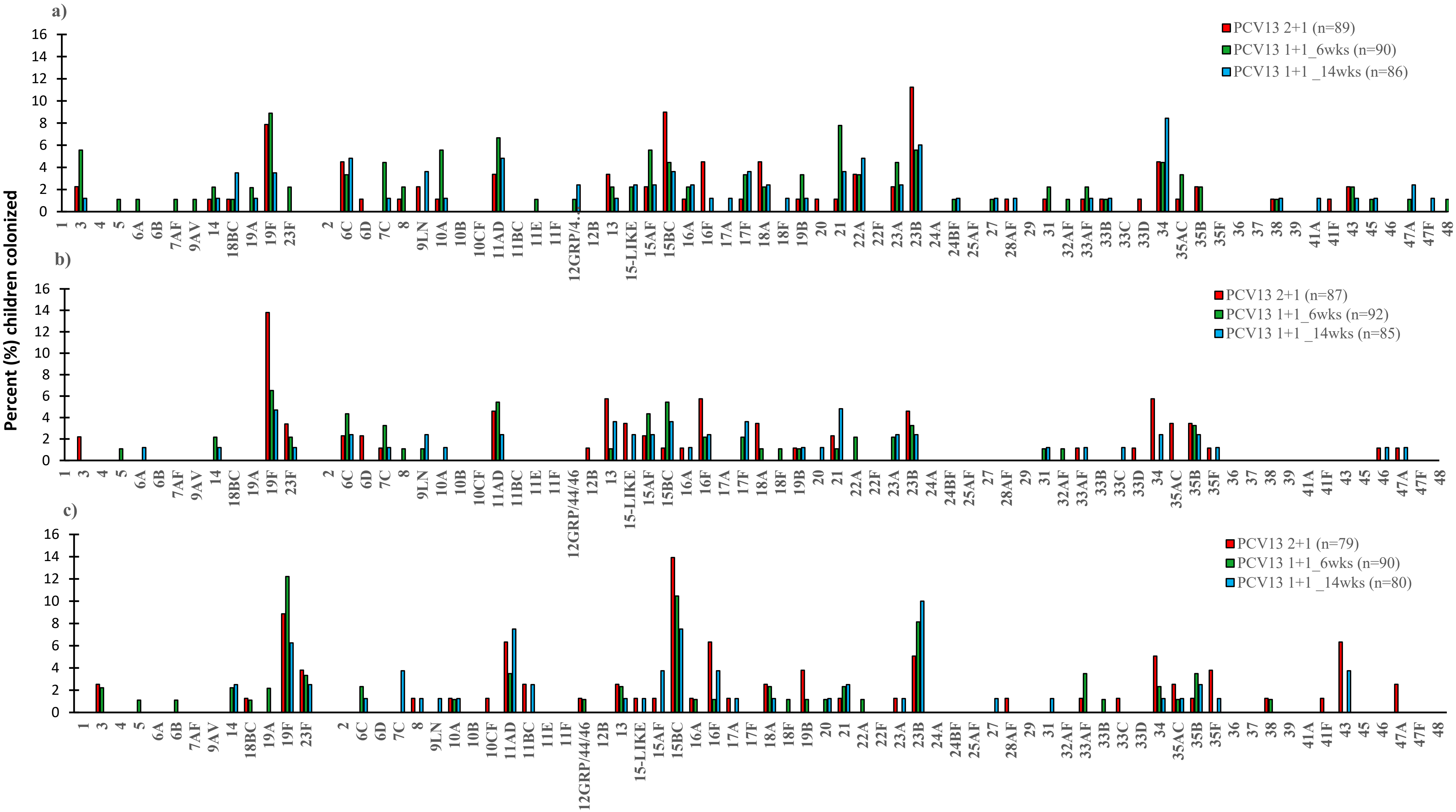

Supplementary Figure 3: Prevalence of pneumococcal serotypes stratified by different PCV13 dosing schedules at a) 9 months of age; b) 15 months of age; c) 18 months of age

Supplementary Table 4: Incidence of new acquisition of vaccine, non-vaccine serotypes and vaccine serotype 19F[1]

Time	*Type*	*PCV13*					*PCV10*				
		2+1	14w+1	*p-value[2]*	6w+1	*p-value[3]*	*2+1*	*14w+1*	*p-value[2]*	6w+1	*p-value[3]*
15m	*VT*	10 (13.2)	5 (6.7)	0.276	4 (6.1)	0.259	8 (10.3)	4 (6.5)	0.549	10 (13)	0.625
15m	*NVT*	12 (34.3)	13 (31)	0.81	6 (20)	0.269	16 (32.7)	7 (18.9)	0.219	10 (21.3)	0.254
15m	*19F*	7 (8.9)	2 (2.5)	0.098	2 (2.5)	0.098	4 (4.9)	1 (1.5)	0.378	6 (7.6)	0.532
18m	*VT*	7 (11.7)	7 (10.6)	>0.999	11 (18)	0.445	10 (14.7)	7 (12.5)	0.797	5 (7.7)	0.275
18m	*NVT*	8 (40)	8 (29.6)	0.541	7 (29.2)	0.532	8 (25.8)	13 (44.8)	0.176	11 (31.4)	0.786
18m	*19F*	4 (6.2)	3 (4.1)	0.705	4 (5.2)	1	7 (9.3)	7 (10.9)	0.784	4 (5.6)	0.534
9 to 18m	*VT*	17 (22.4)	12 (16)	0.409	15 (22.7)	1	18 (23.1)	11 (17.5)	0.53	15 (19.5)	0.695
9 to 18m	*NVT*	20 (57.1)	21 (50)	0.647	13 (43.3)	0.324	24 (49)	20 (54.1)	0.669	21 (44.7)	0.688
9 to 18m	*19F*	11 (13.9)	5 (6.2)	0.122	7 (7.5)	0.21	11 (13.6)	8 (11.8)	0.809	10 (12.7)	>0.999

[1]Values shown are number of events (events per 100 children)
[2]Comparing 2+1 to 14w+1
[3]Comparing 2+1 to 6w+1

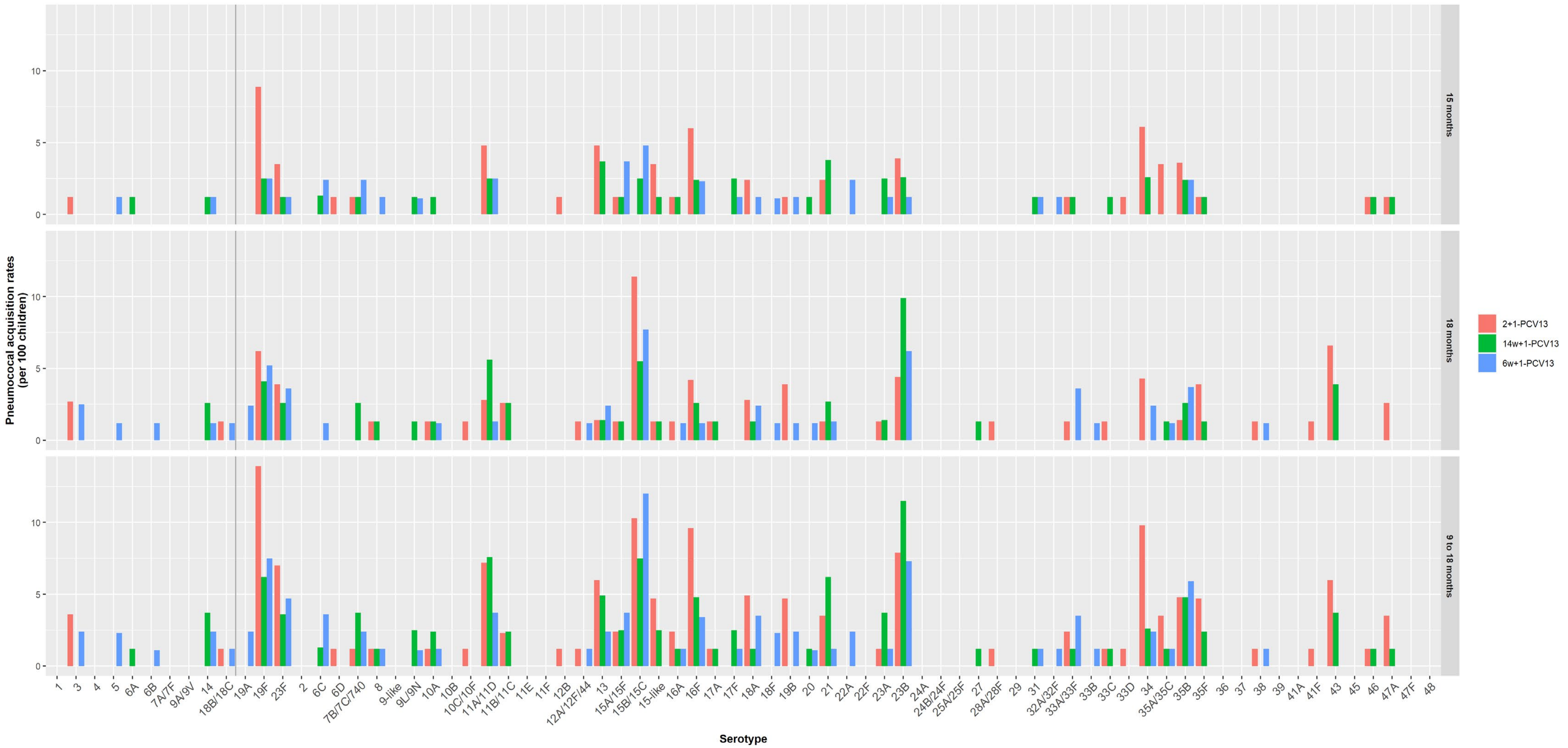

Supplementary Figure 4: Serotype-specific acquisition of new serotypes in children receiving alternative doses of PCV13 at a) 15 months-of-age, 18 months-of-age, and between 9 and 18 months-of-age

New acquisition of a pneumococcal serotype was defined as nasopharyngeal colonization by a serotype not identified at 9 months-of-age and identified at 15 or 18 months-of-age. The pneumococcal acquisition rates were expressed per 100 children.

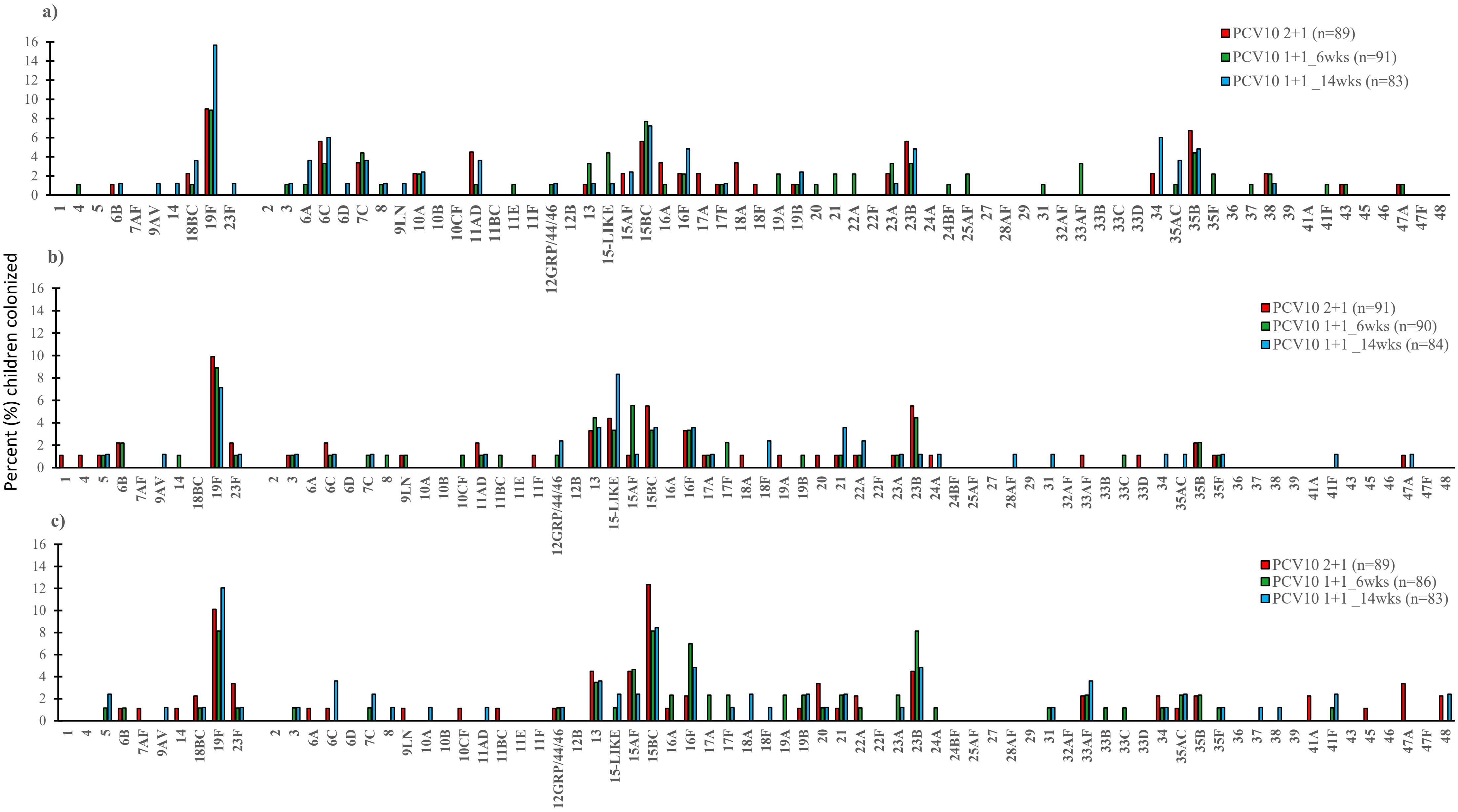

Supplementary Figure 5: Prevalence of pneumococcal serotypes stratified by different PCV10 dosing schedules at a) 9 months of age; b) 15 months of age; c) 18 months of age

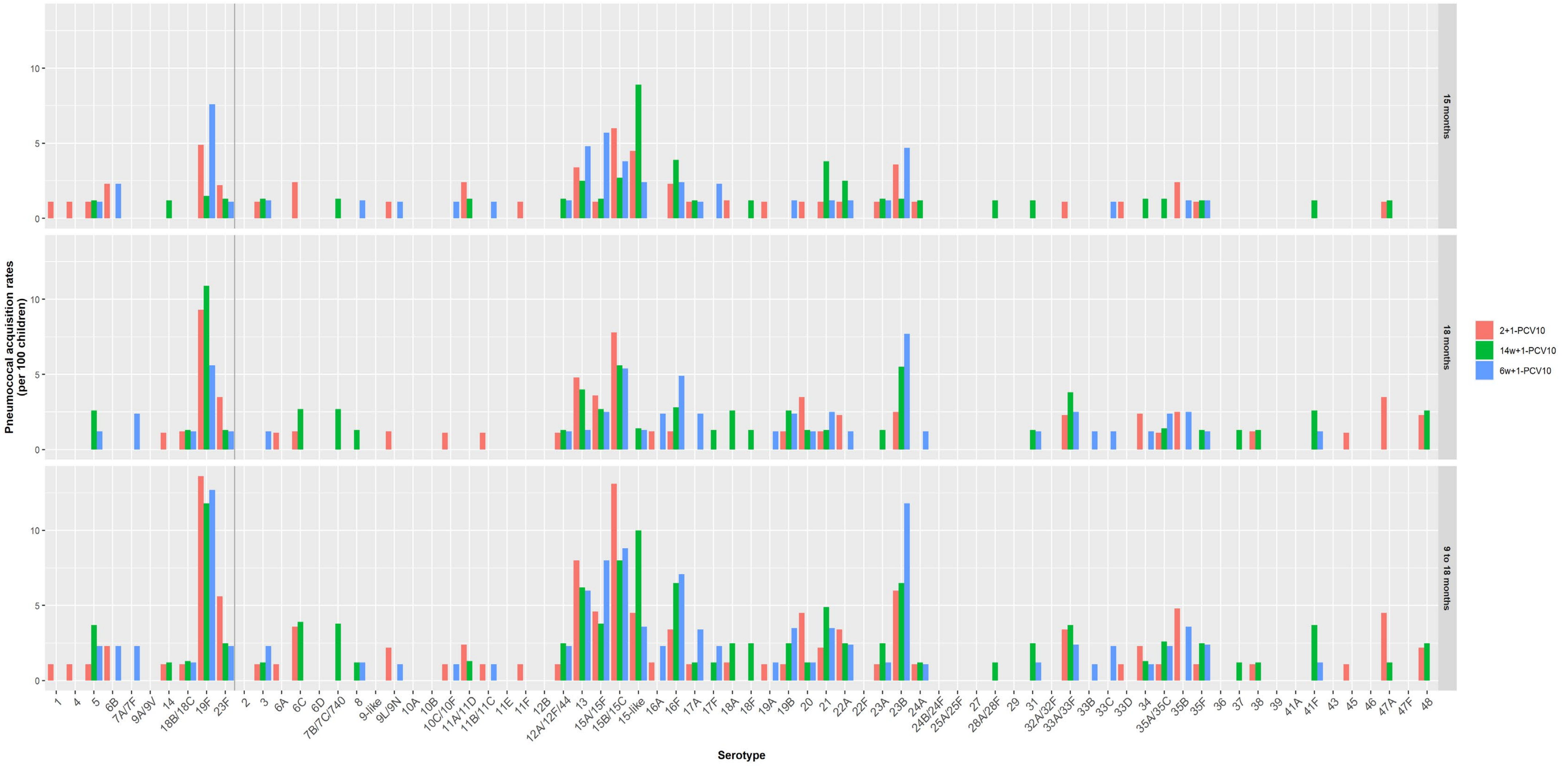

Supplementary Figure 6: Serotype-specific acquisition of new serotypes in children receiving alternative doses of PCV10 at a) 15 months-of age, 18 months-of age, and between 9 and 18 months of age

New acquisition of a pneumococcal serotype was defined as nasopharyngeal colonization by a serotype not identified at 9 months-of-age and identified at 15 or 18 months-of-age. The pneumococcal acquisition rates were expressed per 100 children.

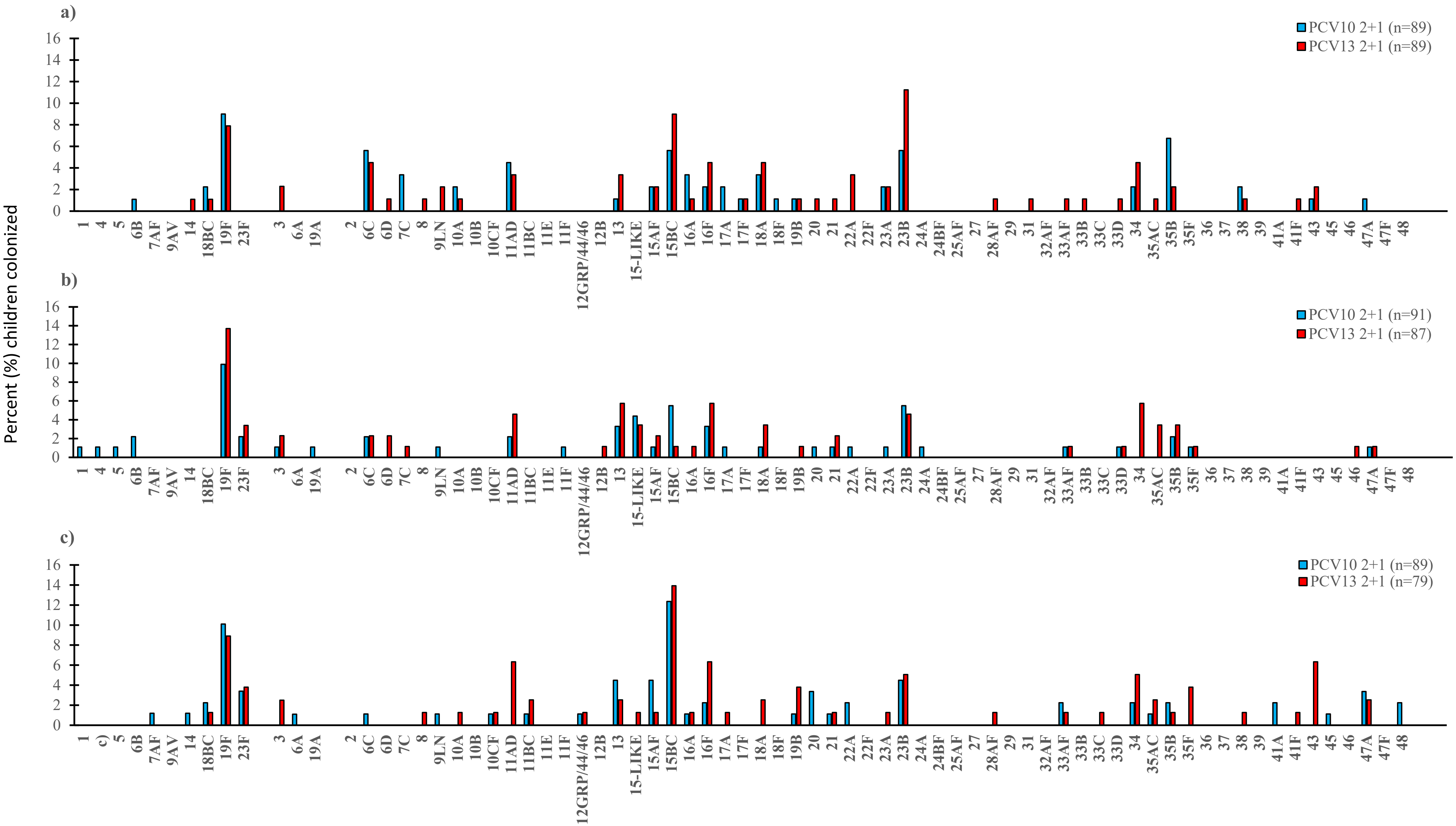

Supplementary Figure 7: Prevalence of pneumococcal serotypes stratified by different PCV formulations at a) 9 months of age; b) 15 months of age and c) 18 months of age
